# Carotid Reservoir Pressure Decrease After Prolonged Head Down Tilt Bed Rest in Young Healthy Subjects Is Associated With Reduction in Left Ventricular Ejection Time and Diastolic Length

**DOI:** 10.3389/fphys.2022.866045

**Published:** 2022-03-25

**Authors:** Carlo Palombo, Michaela Kozakova, Carmela Morizzo, Lorenzo Losso, Massimo Pagani, Paolo Salvi, Kim H. Parker, Alun D. Hughes

**Affiliations:** ^1^Department of Surgical, Medical, Molecular Pathology and Critical Area Medicine, University of Pisa, Pisa, Italy; ^2^Department of Clinical and Experimental Medicine, University of Pisa, Pisa, Italy; ^3^Department of Medical Toxicology Unit and Poison Control Centre, Careggi University Hospital, Florence, Italy; ^4^Department of Medicine, University of Milan, Milan, Italy; ^5^Department of Cardiology, Istituto Auxologico Italiano, IRCCS, Milan, Italy; ^6^Department of Bioengineering, Imperial College London, London, United Kingdom; ^7^Department of Population Science and Experimental Medicine, University College of London, London, United Kingdom

**Keywords:** head-down tilt bed rest, arterial pressure waveform, reservoir pressure, excess pressure, forward pressure wave, backward pressure wave, Windkessel function, systemic haemodynamics

## Abstract

**Background:**

The arterial pressure waveform reflects the interaction between the heart and the arterial system and carries potentially relevant information about circulatory status. According to the commonly accepted ‘wave transmission model’, the net BP waveform results from the super-position of discrete forward and backward pressure waves, with the forward wave in systole determined mainly by the left ventricular (LV) ejection function and the backward by the wave reflection from the periphery, the timing and amplitude of which depend on arterial stiffness, the wave propagation speed and the extent of downstream admittance mismatching. However, this approach obscures the ‘Windkessel function’ of the elastic arteries. Recently, a ‘reservoir-excess pressure’ model has been proposed, which interprets the arterial BP waveform as a composite of a volume-related ‘reservoir’ pressure and a wave-related ‘excess’ pressure.

**Methods:**

In this study we applied the reservoir-excess pressure approach to the analysis of carotid arterial pressure waveforms (applanation tonometry) in 10 young healthy volunteers before and after a 5-week head down tilt bed rest which induced a significant reduction in stroke volume (SV), end-diastolic LV volume and LV longitudinal function without significant changes in central blood pressure, cardiac output, total peripheral resistance and aortic stiffness. Forward and backward pressure components were also determined by wave separation analysis.

**Results:**

Compared to the baseline state, bed rest induced a significant reduction in LV ejection time (LVET), diastolic time (DT), backward pressure amplitude (bP) and pressure reservoir integral (INTPR). INTPR correlated directly with LVET, DT, time to the peak of backward wave (bT) and stroke volume, while excess pressure integral (INTXSP) correlated directly with central pressure. Furthermore, Δ.INTPR correlated directly with Δ.LVET, and Δ.DT, and in multivariate analysis INTPR was independently related to LVET and DT and INTXSP to central systolic BP.

**Conclusion:**

This is an hypothesis generating paper which adds support to the idea that the reservoir-wave hypothesis applied to non-invasively obtained carotid pressure waveforms is of potential clinical usefulness.

## Introduction

The arterial blood pressure (BP) waveform reflects the complex interaction between the heart and the arterial system and carries potentially relevant information about circulatory status (Mynard et al., 2020). According to the commonly accepted ‘wave transmission model’, the net BP waveform results from the super-position of discrete forward (incident) and backward (reflected) traveling pressure waves, with the forward wave in systole determined mainly by the left ventricular (LV) ejection function and the backward wave by the wave reflection from the periphery, the timing and amplitude of which depend on arterial stiffness, the wave propagation speed and the extent of downstream admittance mismatching (Westerhof et al., 1972). This approach, however, obscures the cushioning effect of the elastic arteries, the reservoir function (commonly referred to as the ‘Windkessel function’). It is also worth noting that variables derived from pressure waveform analysis based solely upon the wave transmission model, while predicting cardiovascular events, give little information about arterial function (Westerhof and Westerhof, 2013).

More recently Wang et al. described a mathematical model which includes the ‘Windkessel’ function in shaping the central arterial waveform, combining the wave-only theory (which explains the steep systolic pressure upstroke) with the arterial reservoir, which accounts for the diastolic decline (Wang et al., 2003).

This alternative model has been termed as ‘reservoir-wave’ or ‘reservoir-excess pressure’ (Davies et al., 2007) and interprets arterial BP waveform as a composite of a volume-related, ‘reservoir’ pressure, and a wave-related ‘excess’ pressure which, in turn, can be decomposed into incident and reflected waves (Hughes et al., 2012).

The model has been recently refined, highlighting the concept that both the reservoir and excess pressure are wave phenomena (Hughes and Parker, 2020).

Reservoir pressure has been shown to be highly correlated with changes in proximal aortic volume in the dog (Wang et al., 2003) as well as in man (Schultz et al., 2014), confirming the hypothesis that it represents the cyclic volume increase and decrease during systole and diastole, i.e., of aortic distension and recoil. The aortic reservoir pressure is proportional to the volume of blood stored in the aorta, which in turns depends on the compliance of the aorta and the impedance to outflow (Davies et al., 2010). The ‘excess’ pressure is defined as the difference between the measured and the reservoir pressure and it is assumed to be the result of local waves (Parker et al., 2012). Consequently, the central BP during systole can be considered to be the result of forward wave propagation (as result of LV ejection) and the proximal aortic reservoir function (Davies et al., 2010), while the reservoir pressure will account for almost all the pressure recorded in diastole (Alastruey, 2010).

Despite a growing body of evidence indicating that the reservoir-excess pressure model may provide physiological and clinical insights above and beyond standard BP and pulse waveform analysis (Armstrong et al., 2021) and explain some unsolved issues of the simple wave propagation model, such as the presence of a definite diastolic component of the pressure waveform when net wave travel is close to zero (Parker, 2013), the clinical usefulness of the reservoir-wave paradigm is still debated (Segers et al., 2012).

Beyond some methodological reservations, recently overcome (Hughes and Parker, 2020), a main limitation of this approach for extended clinical use was that it initially required measurement of aortic flow to calculate reservoir pressure in systole. The development of an algorithm which enables reservoir pressure to be calculated from any pressure waveform alone (Michail et al., 2018) opens new horizons to the investigation of the reservoir-excess pressure model in the clinical setting.

In the present study, we applied the reservoir-excess pressure approach to the analysis of carotid arterial pressure waveforms in young healthy volunteers before and after prolonged bed rest which, as we have previously reported, induced a significant reduction in stroke volume (SV), end-diastolic LV volume and LV longitudinal function without significant changes in central blood pressure, cardiac output, total peripheral resistance and aortic stiffness (Kozàkovà et al., 2011; Palombo et al., 2015). In this model, we reassessed arterial pressure waveforms derived by applanation tonometry together with echocardiographic data in order to evaluate the haemodynamic determinants of the aortic reservoir and excess pressure components.

## Materials and Methods

### Study Population

Ten healthy young volunteers, all men, mean age 23 ± 2 years, were enrolled in a bed rest study endorsed by the Italian Space Agency (ASI) and taking place at the Orthopedic Hospital Valdoltra, Ankaran, Slovenia. None of the volunteers was a smoker. Medical history, physical examination, laboratory examinations, resting and stress ECG and echocardiography have excluded any acute or chronic medical problem. The National Committee for Medical Ethics of the Slovene Ministry of Health (Ljubljana, Slovenia) approved the study. All participants were informed about the aim of the investigation, the procedures and the methods and signed a written informed consent form according to the Declaration of Helsinki.

### Study Protocol

All participants underwent a 5-week period of bed rest in a 6°-head down tilt position (HDTBR). The study design and protocol were previously reported in detail (Kozàkovà et al., 2011; Palombo et al., 2015). Cardiac ultrasound, carotid applanation tonometry and carotid–femoral pulse wave velocity (cf-PWV) were performed the day before entering bed rest and within 24 h after its termination. All the examinations were performed by a single operator (CM) in a quiet room, 3 h after a light breakfast and, in case of post-bed rest examination, after an acclimatisation period of 30 min in a supine position.

### Cardiac and Vascular Measurements

Cardiac ultrasound was performed as previously described (Kozàkovà et al., 2011). Stroke volume was measured as a product of aortic area and flow velocity integral in the aortic orifice (Lewis et al., 1984). Total arterial compliance (TAC) was estimated as the ratio of stroke volume to central pulse pressure (SV/cPP; Liu et al., 1986). Results on changes in LV volume mass, performance and loading conditions observed in the same study group were previously published in detail (Kozàkovà et al., 2011). Cf-PWV was measured according to current guidelines (Reference Values for Arterial Stiffness’ Collaboration, 2010) using the Complior SP device (Alam Medical, Vincennes, France). Briefly, arterial waveforms were obtained transcutaneously over the right common carotid artery (CCA) and the femoral artery, and the time delay (*t*) was measured between the feet of the two waveforms. The distance (*D*) covered by the waves was established as the distance between the two recording sites. Cf-PWV was then calculated as *D* (meters)/*t* (seconds). The measurement was repeated three times and the mean value was used for statistical analysis.

Simultaneous BP measurement was performed at the left brachial artery (Omron, Kyoto, Japan).

Carotid applanation tonometry was performed on the right CCA using a PulsePen device (DiaTecne, San Donato Milanese, Italy; Salvi et al., 2004). Carotid pressure waveforms were calibrated according to brachial mean and diastolic pressure as previously described (Van Bortel et al., 2001). For each study, three consecutive acquisitions of 10 cardiac beats were performed, and the mean of three ensemble-averaged cycles was used for statistical analysis. The ensemble-averaged carotid pressure waveform was also decomposed into a forward and backward pressure wave (fP and bP, respectively), as previously described (Qasem and Avolio, 2008). From the carotid pressure waveform, the following parameters were measured, without the application of a generalised transfer function: local systolic BP, local pulse pressure, augmentation index (AIx), pressure at the inflection point (Pi), left ventricular ejection time (LVET), diastolic time (DT), R–R interval, forward and backward wave pressure amplitudes (fP and bP, respectively) and time to the forward and backward pressure wave peak (fT and bT, respectively).

### Reservoir and Excess Pressure Analysis

Reservoir and excess pressure parameters were calculated based on a pressure-alone approach from the ensemble-averaged carotid pressure waveforms, which can be separated in a reservoir pressure component and an excess pressure component, represented by the difference between the measured pressure waveform and the reservoir pressure (Michail et al., 2018). For analysis in this study, we used the integrals of the reservoir pressure curves (INTPR) and excess pressure curves (INTXSP).

### Statistical Analysis

Quantitative data are expressed as mean ± SD or number (%). Skewed data (AIx) are expressed as median [interquartile range] and were log-transformed for statistical analysis. A paired *t*-test was used to compare the measurements before and after bed rest. Linear correlation analysis was used to evaluate the associations of INTPR, INTXPR, fP, bP, cf-PWV and TAC with systemic haemodynamics parameters. A multivariate analysis was performed to evaluate the independent determinants of INTPR, INTXPR, fP, bP. All analyses were adjusted for study phase, age and anthropometric data. Statistical analysis was performed by JMP software, version 16.0.0 (SAS Institute Inc., Cary, North Carolina, United States), and statistical significance was set at a value of <0.05.

## Results

### Impact of Bed Rest on Carotid Pressure Waveform, Systemic Haemodynamics, Regional and Systemic Stiffness

Data obtained by applanation tonometry, echocardiography and carotid–femoral pulse wave measurements are reported in Table 1. When compared to the baseline state, bed rest induced a significant reduction in R–R interval, LVET, DT, fT and bP, a non-significant decrease in fP as well in central BP and no changes in AIx, cf-PWV, TAC. Bed rest also significantly decreased INTPR, while the reduction of INTXSP was only mild and non-significant.

**Table 1:** Haemodynamic data obtained by applanation tonometry, echocardiography and carotid–femoral pulse wave velocity measurements before and after the bed rest.

	Before bed rest	After bed rest	*p*
BMI (kg/m^2^)	23.3 ± 2.0	22.8 ± 1.6	<0.05
Central systolic BP (mmHg)	106 ± 12	101 ± 7	ns
Central pulse pressure (mmHg)	43 ± 11	36 ± 7	ns
Heart rate (bpm)	59 ± 8	71 ± 8	<0.0001
R–R (ms)	1,030 ± 171	851 ± 78	<0.01
LVET (ms)	305 ± 20	292 ± 11	<0.05
DT (ms)	723 ± 158	559 ± 75	<0.01
AIx (%)	2.0 [8.1]	2.1 [8.5]	ns
Pi (mmHg)	104 ± 13	100 ± 6	ns
fP (mmHg)	42.1 ± 12.3	35.1 ± 7.1	0.06
bP (mmHg)	13.0 ± 2.9	10.8 ± 2.3	0.05
fT (ms)	102 ± 25	90 ± 12	<0.05
bT (ms)	316 ± 86	268 ± 33	ns
INTPR (kPa*s)	10.6 ± 1.7	9.0 ± 1.3	<0.01
INTXSP (kPa*s)	0.77 ± 0.28	0.64 ± 0.25	ns
End-diastolic LV volume (ml)	144 ± 15	131 ± 18	<0.005
EF (%)	58 ± 3	57 ± 4	ns
Stroke volume (ml)	74 ± 10	62 ± 8	<0.01
Cardiac output (L/min)	4.6 ± 0.8	4.5 ± 0.6	ns
TAC (ml/mmHg)	1.85 ± 0.55	1.72 ± 0.32	ns
cf-PWV (m/ss)	6.9 ± 1.0	6.6 ± 0.8	ns

**Figure 1:**
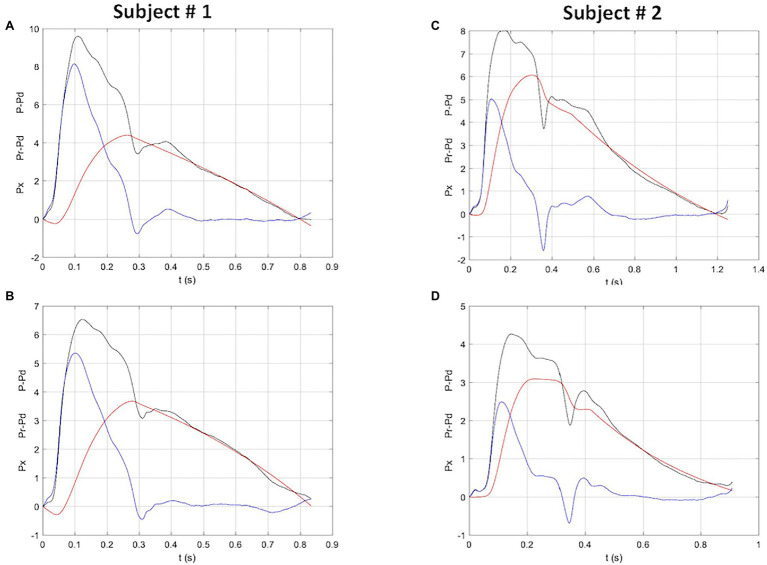
Reservoir, excess and net pressure waveforms (red, blue and black, respectively), in two study subjects before (**top panels**) and after (**bottom panels**) bed rest. Both subjects show a reduction in net, reservoir and excess pressure. Subject # 2 also shows a clear reduction of the time to peak reservoir pressure. P-Pd, peak systolic pressure minus diastolic pressure; Pr-Pd, reservoir pressure minus diastolic pressure; Px, excess pressure. Units: *X*-axis, seconds; *Y*-axis, kPa.

Figure 1 shows an example of net, reservoir and excess pressure before and after bed rest. Within the echocardiographic parameters, stroke volume decreased with bed rest, while cardiac output did not change due to the increase in heart rate.

### Univariate Correlations

Table 2 shows correlation coefficients of the associations between INTPR, INTXSP, fP, bP, TAC, cf-PWV and the systemic haemodynamics, echocardiographic data, systolic and diastolic times. In analyses were included data obtained both before and after bed rest (*N* = 20). Values reported are after adjustment for study phase. INTPR correlated directly with R–R interval, LVET, DT, stroke volume and TAC. INTXSP correlated directly with central pressure, systolic and pulse, Pi and fP and inversely with TAC. Backward pressure amplitude correlated directly with R–R interval, DT, forward pressure amplitude and stroke volume. TAC correlated directly with time to the forward pressure peak, while direct correlations were found for cf-PWV with stroke volume and TAC.

**Table 2:** Correlation coefficients of reservoir pressure, excess pressure, separated backward and forward pressures, total arterial compliance and aortic stiffness with systemic haemodynamics parameters.

	INTPR (kPa*s)	INTXSP (kPa*s)	fP (mmHg)	bP (mmHg)	TAC (ml/mmHg)	cfPW-V (m/s)
cSBP (mmHg)	−0.01	0.66^**^	–	–	–	−0.06
cPP (mmHg)	−0.01	0.61^**^	–	–	–	−0.13
R–R (ms)	0.88^**^	−0.11	0.01	0.46^*^	0.33	0.13
LVET (ms)	0.74^**^	−0.34^*^	−0.29	0.05	0.41	−0.02
DT (ms)	0.86^**^	−0.08	0.03	0.49^*^	0.30	0.13
AIx (%)	0.21	−0.30	–	–	0.40	0.19
Pi (mmHg)	−0.03	0.68^**^	–	–	–	−0.09
fP (mmHg)	−0.15	0.62^**^	–	0.69^**^	–	−0.15
bP (mmHg)	0.40	0.40	0.69^**^	–	–	−0.07
fT (ms)	0.48	−0.27	−0.49^**^	−0.07	0.60^**^	0.44
bT (ms)	0.45	−0.17	−0.05	−0.2	0.13	0.07
EDV (ml)	0.45	−0.20	0.04	0.41	–	−0.15
SV (ml)	0.60^*^	0.11	0.28	0.45^*^	–	0.47^*^
TAC (ml/mmHg)	0.45^*^	−0.49^*^	–	–	–	0.48^*^

** *p* *at least < 0.01;*

* *p < 0.05*.

The relationships between bed rest related changes (delta) in different parameters were also evaluated. Delta INTPR correlated directly with delta LVET (*r* = 0.85; *p* < 0.005) and delta DT (*r* = 0.68; *p* < 0.05). Delta fP correlated with delta bP (*r* = 0.81; *p* < 0.005) as well as with change in central pulse pressure (*r* = 0.82; *p* < 0.005).

### Multivariate Analysis

Table 3 demonstrates independent determinants of INTPR, INTXSP, wave pressure amplitudes and aortic stiffness. INTPR was independently related to LVET and DT and INTXSP to central systolic BP. The only independent predictor of fP was BMI, while bP was independently related to DT and fP.

**Table 3:** Independent determinants of reservoir and excess pressure and separated pressure wave amplitudes.

	*b* ± SE	*p*
INTPR (kPa*s)		
LVET (ms)	0.38 ± 0.12	<0.0001
DT (ms)	0.68 ± 0.14	<0.05
Cumulative *R*^2^	0.83	<0.0001
*INTXSP*		
Central SBP (mmHg)	0.65 ± 0.18	0.001
Cumulative *R*^2^	0.45	0.001
*fP (mmHg)*		
BMI (kg/m^2^)	0.60 ± 0.18	<0.005
Cumulative *R*^2^	0.48	<0.005
*bP (mmHg)*		
DT (ms)	0.55 ± 0.17	<0.005
fP (mmHg)	0.73 ± 0.15	0.0001
Cumulative *R*^2^	0.71	<0.0001

## Discussion

The analysis of central arterial pressure waveform as a method providing relevant information about the interaction between LV and arterial function is gaining increasing clinical interest thanks to the availability of methods capable of capturing the signal in an accurate non-invasive way (Salvi et al., 2004). Although it is commonly assumed that arterial pressure waveforms are purely the result of forward and backward traveling waves, it has been pointed-out that arterial behaviour is difficult to explain using this assumption, particularly during the diastolic phase (Parker, 2013). Thus, a ‘reservoir-wave’ hypothesis has been introduced as a heuristic model, which gives emphasis to the role of aortic compliance (‘reservoir’ or ‘Windkessel’) and the change in arterial volume over the cardiac cycle, although preserving an important role for wave reflections and re-reflections in shaping the morphology of pressure (and flow) waveforms (Hughes et al., 2012; Hughes and Parker, 2020). This ‘hybrid’ reservoir-wave (or ‘reservoir-excess pressure’) model, is based upon the premise that not all changes in aortic pressure can be ascribed to forward and backward traveling waves and interprets the arterial BP waveform as a composite of a volume-related, ‘reservoir’ pressure, and a wave-related (‘excess’) pressure. It is thought to better describe the pressure waveform, providing physiological and clinical insights above and beyond the standard BP and pulse waveform analysis (Armstrong et al., 2021). Furthermore, reservoir and excess pressure analysis was recently shown to provide prognostically useful markers in various patients populations (Davies et al., 2014; Hametner et al., 2014; Narayan et al., 2015; Aizawa et al., 2021).

Nonetheless, doubts and reservations about the methodologic foundation and potential clinical usefulness of the ‘reservoir-wave’ approach still persist (Segers et al., 2012; Mynard and Smolich, 2014).

In this work, we aimed to verify the haemodynamic determinants of the aortic reservoir and excess pressure components, as well as their relations with forward and backward pressure waves, in a unique clinical model; the prolonged head down tilt bed rest (HDTBR). HDTBR mimics microgravity conditions and represents an established model of chronic circulatory unloading associated with a significant decrease in total body water and stroke volume, together with a parallel adaptive reduction in longitudinal LV myocardial function and a compensatory relative tachycardia, resulting in unchanged cardiac output (Kozàkovà et al., 2011).

### Impact of Prolonged HDTBR on Carotid Reservoir and Wave Pressure Components

In 10 young healthy volunteers, the reservoir pressure integral (INTPR) significantly decreased after prolonged bed rest, together with significant reductions in SV, R–R interval, left ventricular ejection time (LVET) and diastolic time (DT); the amplitude of backward pressure (bP) and the time to the peak of forward pressure (fT) were also significantly reduced. Reductions at the limits of statistical significance were found for central pulse pressure (cPP) and amplitude of forward pressure (fP), while no significant changes were observed for the excess pressure integral (INTXSP), augmentation index (AIx), aortic stiffness (cf-PWV) and total arterial compliance.

### Reservoir and Excess Pressure Integrals and Their Physiologic Determinants

Pooling together individual data obtained before and after bed rest, we observed significant direct correlations between INTPR and SV, as well as between INTXSP and all systolic pressure components (cSBP, cPP, fP). Furthermore, INTPR was directly correlated also with LVET and DT. Taken together, these findings are substantially in keeping with the previous demonstrations by Schultz et al. (2014) that the reservoir pressure reflects the volume of blood transiently stored in the aorta soon after the ejection, by Hametner et al. (2014) of a direct relation between reservoir pressure and SV and heart rate, and the observation of Parker (2013) that the reservoir pressure will be primarily dependent upon heart rate and the global arterial properties. In our study, these conclusions are further supported by the finding of LVET and DT as independent predictors of the reservoir pressure integral and the observation of direct significant correlations between changes (Δ) in INTPR after bed rest compared to baseline and the corresponding changes in LV ejection time and diastolic time, in keeping with the hypothesis that reservoir pressure mainly depends on the LV ejection function during systole and on the aortic Windkessel during diastole (Armstrong et al., 2021). On the other hand, the correlations observed between excess pressure integral (INTXSP) and central BP (systolic and pulse) and amplitude of forward pressure component, without any association with backward pressure amplitude or AIx, supports the hypothesis that it mainly reflects, at least in normal subjects, the early systolic LV pump function (Parker, 2013).

### Separated Pressure Wave Analysis

Worth noting are the observations, in our study, of a robust direct association between the amplitude of forward and backward pressure waves, as well as between the reflected wave amplitude and R–R period and diastolic time. Thus, in conditions of normal arterial stiffness associated to young age, a main determinant of backward pressure amplitude seems to be the forward pressure amplitude, as previously reported by our group in different patients using a wave intensity approach (Rakebrandt et al., 2009).

### Pulse Wave Velocity and Total Arterial Compliance

The direct association we observed between cf-PWV and stroke volume and time to the forward pressure peak (fT) is apparently counterintuitive and in contrast with the wave model, according to which cf-PWV represents aortic stiffness leading to an inverse relation between cf-PWV and fT. Actually, in young arteries with normal stiffness, even the speed of wave propagation seems to be related to haemodynamic variables, such as the volume flow (i.e., stroke volume).

### Impact of Heart Rate in Shaping the Arterial Waveform

In our work, the R–R period and its systolic and diastolic segments were independent determinants of both reservoir pressure and the amplitude of the backward pressure wave. These findings are in agreement with those of Hametner et al. (2014), who showed highly significant relations of reservoir pressure with heart rate (inverse) and backward pressure amplitude (direct).

### ‘Reservoir/Excess Pressure’ and ‘Wave-Alone’ Model: Not Alternative but Complementary

Our data, together with those from the literature (Parker, 2013; Hametner et al., 2014; Hughes and Parker, 2020; Armstrong et al., 2021), tend to support the clinical usefulness of the reservoir-excess pressure model in interpreting physiological phenomenon and arterial pressure waveform features not completely explained by the wave-alone model. This is not alternative but complementary to the commonly accepted wave model, which considers the arterial waveform as the result of a super-position of a backward reflected wave. The forward wave is the result of the coupling between the activity of the left ventricle and the viscoelastic properties of the aorta (Windkessel phenomenon), while the backward waves are the result of the multiple reflections of the single waves, affected therefore by numerous factors (peripheral vascular resistance, distance of the reflection sites, heart rate). Both forward and backward waves are affected by arterial stiffening.

Integrating the Windkessel function into the model (Davies et al., 2007), the reservoir pressure will account for the systolic pressure associated with the blood inflow entering the aorta as well as for almost all the pressure recorded in diastole (Parker et al., 2012). For a given arterial stiffness, at least in young normal subjects, LV ejection time and diastolic time are key determinants of the pressure waveform features, and backward reflected wave occurring in the diastolic phase may contribute to support reservoir and diastolic pressure, instead of increasing LV systolic load as expected with an increased large artery stiffness.

### Study Limitations

This study involves a small and selected population, represented by young healthy subjects, and thus, our findings cannot be extrapolated to older subjects with risk factors and increased arterial stiffness. Furthermore, correlation analyses were performed including individual data acquired both before and after bed rest. However, we accounted for the lack of independence in variables within an individual.

## Conclusion

This is a confirmatory study, reproducing previous findings in an original ‘clinical’ model. However, according to Armstrong et al. (2021), ‘the understanding of the reservoir-excess pressure model is still in its relative infancy, and more research is needed’. The paper provides a piece of evidence supporting the idea that the reservoir-wave hypothesis applied to non-invasively obtained carotid pressure waveforms is of potential clinical usefulness. Our results confirm the hypothesis that reservoir—excess pressure analysis complements the wave model adding a volume (‘Windkessel’) component, which may have variable impact and relevance according to age and health/disease status of the subject. In perspective, the pressure waveform analysis performed combining the two models with an assessment of the haemodynamic status by echocardiography and an estimate of aortic stiffness would allow us to disentangle the role of various mechanisms in shaping the pressure waveform, with the aim of defining the informative content of the pressure waveform for clinical purposes in the individual subjects.

## Data Availability Statement

The raw data supporting the conclusions of this article will be made available by the authors, without undue reservation.

## Ethics Statement

The studies involving human participants were reviewed and approved by The National Committee for Medical Ethics of the Slovene Ministry of Health (Ljubljana, Slovenia). The patients/participants provided their written informed consent to participate in this study.

## Author Contributions

CP contributed to the conceptualisation, data analysis, manuscript drafting and editing. AH, KP and PS developed the analytical methods and critically revised the manuscript. MK and MP contributed to the conceptualisation, manuscript editing, and project supervision. CM and LL contributed to the data acquisition and management. All authors contributed to the article and approved the submitted version.

## Funding

This study was partly supported by grants of the Italian Space Agency (ASI), projects Disorders of Motor and Cardio-Respiratory Control (DMCR) and Osteoporosis and Muscle Atrophy (OSMA), by a grant (PRIN 2007) of the Italian Ministry of University and Research (MIUR), and partially supported by the Italian Ministry of Health.

## Conflict of Interest

MK is responsible for clinical studies at Esaote SpA (Genova, Italy). PS is consultant for DiaTecne s.r.l. (San Donato Milanese, Italy).

The remaining authors declare that the research was conducted in the absence of any commercial or financial relationships that could be construed as a potential conflict of interest.

## Publisher’s Note

All claims expressed in this article are solely those of the authors and do not necessarily represent those of their affiliated organizations, or those of the publisher, the editors and the reviewers. Any product that may be evaluated in this article, or claim that may be made by its manufacturer, is not guaranteed or endorsed by the publisher.

## Abbreviations

BP: Blood pressure
cf-PWV: Carotid–femoral pulse wave velocity
AIx: Augmentation index
Pi: Pressure at the inflection point
LVET: Left ventricular ejection time
DT: Diastolic time
fP: Forward wave pressure amplitudes
bP: Backward wave pressure amplitudes
fT: Time to the forward pressure wave
bT: Time to the backward pressure wave
TAC: Total arterial compliance
