# Corrigendum: Carotid reservoir pressure decrease after prolonged head down tilt bed rest in young healthy subjects is associated with reduction in left ventricular ejection time and diastolic length

**DOI:** 10.3389/fphys.2022.990346

**Published:** 2022-08-17

**Authors:** Carlo Palombo, Michaela Kozakova, Carmela Morizzo, Lorenzo Losso, Massimo Pagani, Paolo Salvi, Kim H. Parker, Alun D. Hughes

**Affiliations:** ^1^ Department of Surgical, Medical, Molecular Pathology and Critical Area Medicine, University of Pisa, Pisa, Italy; ^2^ Department of Clinical and Experimental Medicine, University of Pisa, Pisa, Italy; ^3^ Department of Medical Toxicology Unit and Poison Control Centre, Careggi University Hospital, Florence, Italy; ^4^ Department of Medicine, University of Milan, Milan, Italy; ^5^ Department of Cardiology, Istituto Auxologico Italiano, IRCCS, Milan, Italy; ^6^ Department of Bioengineering, Imperial College London, London, United Kingdom; ^7^ Department of Population Science and Experimental Medicine, University College of London, London, United Kingdom

**Keywords:** head-down tilt bed rest, arterial pressure waveform, reservoir pressure, excess pressure, forward pressure wave, backward pressure wave, systemic hemodynamics, windkessel function

In the published article, there was an error in the **Funding** statement. The funding contribution of the Italian Ministry of Health was omitted. The corrected Funding statement appears below:

This study was partly supported by grants of the Italian Space Agency (ASI), projects Disorders of Motor and Cardio- Respiratory Control (DMCR) and Osteoporosis and Muscle Atrophy (OSMA), by a grant (PRIN 2007) of the Italian Ministry of University and Research (MIUR), and partially supported by the Italian Ministry of Health.

The authors apologize for this error and state that this does not change the scientific conclusions of the article in any way. The original article has been updated.

